# Genomic and Histopathological Tissue Biomarkers That Predict Radiotherapy Response in Localised Prostate Cancer

**DOI:** 10.1155/2015/238757

**Published:** 2015-10-04

**Authors:** Anna Wilkins, David Dearnaley, Navita Somaiah

**Affiliations:** ^1^Division of Clinical Studies, The Institute of Cancer Research, London SM2 5NG, UK; ^2^Division of Radiotherapy and Imaging, The Institute of Cancer Research, London SM2 5NG, UK; ^3^Division of Cancer Biology, The Institute of Cancer Research, London SM2 5NG, UK

## Abstract

Localised prostate cancer, in particular, intermediate risk disease, has varied survival outcomes that cannot be predicted accurately using current clinical risk factors. External beam radiotherapy (EBRT) is one of the standard curative treatment options for localised disease and its efficacy is related to wide ranging aspects of tumour biology. Histopathological techniques including immunohistochemistry and a variety of genomic assays have been used to identify biomarkers of tumour proliferation, cell cycle checkpoints, hypoxia, DNA repair, apoptosis, and androgen synthesis, which predict response to radiotherapy. Global measures of genomic instability also show exciting capacity to predict survival outcomes following EBRT. There is also an urgent clinical need for biomarkers to predict the radiotherapy fraction sensitivity of different prostate tumours and preclinical studies point to possible candidates. Finally, the increased resolution of next generation sequencing (NGS) is likely to enable yet more precise molecular predictions of radiotherapy response and fraction sensitivity.

## 1. Introduction

Heterogeneity in tumour biology between prostate tumours results in a varied response to radiotherapy. At present no molecular tissue biomarkers are in routine clinical use to risk-stratify patients with localised prostate cancer. Instead, current management of localised prostate cancer is based upon established clinical risk factors including presenting PSA, clinical or radiological T (tumour) stage, and the total Gleason score. However, estimates of recurrence and survival vary considerably; for example, in the National Comprehensive Cancer Network (NCCN) intermediate risk group biochemical failure at five years following definitive local therapy varies from 2% to 70% [1]. Although new clinical factors have been identified including percentage core positivity and the primary Gleason score [2], there remains an urgent need to incorporate molecular biomarkers predicting radioresistance into treatment decisions. Such biomarkers would enable a personalised prediction of radiotherapy efficacy. If combined with personalised predictors of radiation toxicity including radiogenomic markers [3], both sides of the therapeutic ratio of radiotherapy for localised prostate cancer would be improved.

The lethality of radiotherapy is centred on the creation of chromosomal lesions including DNA double strand breaks (DSB), which are particularly lethal when they cluster in close physical proximity to each other [4]. Cells that are unable to repair this radiation induced DNA damage are likely to undergo programmed cell death via apoptosis or autophagy or alternatively death via mitotic catastrophe [5, 6]. Hypoxia has traditionally been viewed as an important contributor to radioresistance as oxygen reacts with damaged DNA bases created by free radicals thus creating a stable adduct and fixing the damage [7]. More recently, the hypoxic state has also been associated with reduced capacity for DNA repair, increasing genomic instability, and creation of a mutator phenotype [8]. Whilst biomarkers of DNA repair and hypoxia have been shown to predict radioresistance, much broader aspects of tumour biology including cell proliferation, apoptosis, and androgen synthesis have been implicated in treatment failure following radiotherapy. All of these offer considerable potential for improving treatment precision, for example, with personalised dose escalation or concomitant use of systemic agents such as abiraterone.

Another important radiobiological question at present is the radiotherapy fraction size sensitivity of prostate cancer, as measured by the alpha/beta ratio. An expanding body of evidence points to the alpha/beta ratio of prostate adenocarcinoma being as low as 1.5 [9], suggesting that tumours are more sensitive to fraction size than neighbouring normal tissues. The results of randomised clinical trials testing this hypothesis are currently awaited [10]. However, it is highly likely that the alpha/beta ratio and therefore fraction size sensitivity differ between individual prostate tumours, especially as we know that cellular proliferation, DNA repair, hypoxia, and other relevant biological parameters vary considerably. Although an exciting area of research, once again no molecular biomarkers are in clinical use to assess fraction size sensitivity of tumours prior to radiotherapy treatment.

Recent rapid progress in next generation sequencing techniques offers huge potential for personalisation of radiotherapy treatment, despite some of the required technological expertise being currently beyond the scope of most routine pathology laboratories. Other routinely available histopathological techniques such as immunohistochemistry (IHC) or genomic techniques such as fluorescent in situ hybridisation (FISH), comparative genomic hybridisation (CGH), and polymerase chain reaction (PCR) have identified many candidate biomarkers which with further validation could rapidly enter the clinic.

Molecular biomarker development following prostatectomy has progressed at an accelerated pace compared to following radiotherapy due to limited tissue availability with the latter treatment [11]. Critics suggest that diagnostic biopsies do not represent the true biological heterogeneity within the entire prostate gland. However, as image-guided template biopsies become more commonly used, tumour representation in prostate biopsies continues to increase in accuracy. Furthermore, for the foreseeable future, diagnostic biopsies will continue to be the main tumour tissue available to guide radiotherapy stratification. It is important that the above differences in tissue availability do not hinder the development and validation of predictive biomarkers that distinguish benefit from different local treatments for early prostate cancer as this remains a clinical priority.

This paper aims to review biomarkers predicting radiotherapy response in prostate cancer incorporating genomic signatures and individual candidates as well as biomarkers identified by longer established techniques. It does not address microRNA or biomarkers involved in the diagnosis of prostate cancer or prognostication outside of radiotherapy treatment; these were comprehensively reviewed in a recent paper in this journal [12].

## 2. Biomarkers of Radiosensitivity Identified Using Immunohistochemistry (IHC)

IHC enables direct evaluation of protein expression, which is advantageous as proteins are determinants of cellular function. Recent comprehensive genomic and proteomic work suggests that changes in nucleic acid do not necessarily translate to corresponding changes in protein expression [13]. IHC is a technique that is readily available in routine pathology laboratories; tumour histopathology can be correlated with protein expression; hence, tumour dissection is not required. For bulky prostate tumours, sufficient tissue may be present to construct tissue microarrays which facilitates high throughput analysis [14, 15]; however, in intermediate risk localised prostate tumours this approach has recently been shown to be unfeasible due to inadequate numbers of tumour cells [16].

The Radiation Therapy Oncology Group (RTOG) 8610 and 9202 clinical trials of radiotherapy and varying lengths of androgen deprivation for localised prostate cancer have reported several biomarkers predicting outcome using IHC (Table 1). A consistent prediction of survival outcomes has been shown for some candidates and the second trial has validated earlier findings. For example, Ki-67, a well-established marker of cellular proliferation, has consistently predicted biochemical-free survival, local recurrence, and overall survival [17–21]. p53, one of the most commonly mutated tumour suppressor genes with a central role in checkpoint activation, is regulated by the oncogene MDM2. Both genes have shown prediction of prostate cancer outcome in the RTOG studies and elsewhere [17, 22–27]. Low expression of the cyclin dependent kinase inhibitor p16 has also been consistently associated with poor survival outcomes following radiotherapy [28, 29]. As poor outcomes following surgery are predicted by high expression, p16 is one of very few true predictive biomarkers identified to date [11]. Finally increased expression of COX2, a gene with cell cycle modulatory effects as well as antiapoptotic, proangiogenic, and proliferative effects via prostaglandin E2 [30, 31], has also been repeatedly associated with poor survival outcomes [32].

Pollack et al. recently modelled the risk of distant metastases using expression of the above 5 candidates plus the apoptotic proteins Bcl-2 and Bax with competing risks hazard regression, adjusting for age, PSA, the Gleason score, T-stage, and treatment [33]. The resulting model included 4 tissue biomarkers (Ki-67, MDM2, p16, and COX2) and showed a concordance index of 0.77 versus 0.70 without molecular biomarkers, meaning a relative improvement in prediction of distant metastases of 10%. This “immunopanel” is the first known multiplex panel of biomarkers developed using IHC to date in prostate cancer.

The role of hypoxia markers in prognostication following radiotherapy is more controversial. VEGF and HIF1-alpha were not included in the RTOG modelling of risk of distant metastases because an earlier RTOG study failed to demonstrate a significant association of VEGF with any survival outcomes following radiotherapy [34]. However, in two British studies of VEGF and HIF1-alpha, increased expression independently predicted biochemical recurrence [35, 36]. Furthermore, lactate dehydrogenase (LDH), a marker of anaerobic metabolism and an indirect marker of hypoxia, has also been associated with inferior radiotherapy response [37]. Osteopontin (OPN) is a small integrin-binding ligand N-linked glycoprotein (SIBLING) that is thought to be induced by hypoxia [38]. OPN has been associated with reduced survival times in prostate cancer [38], however, was only significant in predicting radiotherapy response on univariate analysis, not when modelling adjusted for other clinical factors [35]. A study of plasma OPN levels in localised prostate cancer indicated that OPN levels did not change in response to radiotherapy [46].

There are several possible reasons for conflicting data regarding hypoxic biomarkers and prediction of radiotherapy response. These include differences in the size of patient cohorts, NCCN risk group, and IHC cut points used to determine high expression of VEGF and HIF1-alpha. Furthermore, our understanding of how hypoxia impacts DNA repair is evolving rapidly. Recent studies suggest that hypoxia induces downregulation of proteins within the DNA double strand break repair pathways of homologous recombination (HR) and nonhomologous end joining (NHEJ) [47–49]. This has implications for radiosensitivity and also provides a mechanism for hypoxia inducing a mutator phenotype as DNA repair downregulation could permit survival and subsequent clonal selection of unrepaired unstable mutant tumour cells [8]. Further work to clarify the role of hypoxic markers in treatment stratification would be of considerable value.

With regard to DNA repair, error prone NHEJ operates in all phases of the cell cycle to repair DNA DSB; DNA PKcs has a key role in NHEJ by forming a synaptic complex bringing the free broken ends of DNA together with other ligating enzymes. Nuclear expression of DNA PKcs using IHC showed an independent association with biochemical recurrence after radiotherapy. However, other NHEJ proteins, also evaluated with IHC, such as Ku70, Ku80, and XRCC4 were not predictive of relapse [45].

The TMPRSS2/ERG fusion is an important cellular rearrangement occurring in 50% of localised prostate tumours and ERG protein expression using IHC has been shown to be a robust surrogate for detecting the gene fusion [50]. Pre-clinical studies have suggested that the gene fusion may be a biomarker of inferior double stranded DNA break repair capacity with important clinical implications [51]. However, the gene fusion was not prognostic for recurrence after radiotherapy when assessed with either IHC or CGH suggesting that it does not affect prostate tumour radiosensitivity [16] (Table 3).

There is thought to be direct cross talk between the EGFR cellular proliferation pathway and DNA repair. This provides a possible biological rationale for the observed correlation between high EGFR expression and poor prognosis following prostate radiotherapy [42]. However, the aetiology of poor survival outcomes with increased EGFR expression is likely to be multifactorial and includes increased cellular proliferation. Protein kinase A type 1 and STAT3 also function in cell proliferation and malignant transformation and have been studied in the RTOG trials where overexpression has been associated with poorer outcomes [39, 40]. However, STAT3 expression only correlated with distant metastases and not with other survival outcomes such as local failure. For protein kinase A, overexpression was associated with a diminished response to long term androgen deprivation therapy (LTAD) and radiation, relative to short term androgen deprivation and radiotherapy suggesting that these patients require alternative treatment escalation to LTAD.

The antiapoptotic protein Bcl-2 and proapoptotic Bax both have key roles in determining cell fate following radiotherapy. Independent prediction of survival outcomes has been demonstrated in some [22, 23, 43], but not all [44, 60], studies to date and further work to clarify their prognostic role is needed. Androgen deprivation is known to cause apoptotic cell death so different use of androgen deprivation within treatment arms of RTOG 8610 and 9202 is likely to have impacted predictive outcomes [44]. Some of the observed discrepancies may have arisen due to high numbers of patients with locally advanced versus early prostate cancer, a lack of standardised IHC protocol and antibody, as well as use of different cut points defining high or low expression between studies.

Identifying biomarkers of radiotherapy fraction size sensitivity is another area of unmet need. There is a tight association between proliferative indices and fraction size sensitivity of normal tissues [61]. Normal tissues that respond early (within days of radiotherapy) have high proliferative indices and low sensitivity to fraction size and vice versa for those that respond late (years later). The association is so tight as to offer clues to mechanisms [62–64]. Published literature also suggests that the choice of DNA DSB repair pathway (homologous recombination (HR) versus NHEJ) between rapidly proliferating and slowly proliferating cells may influence fraction size sensitivity. Using* in vitro* clonogenic assays rodent cell lines with defects in NHEJ showed loss of fraction size sensitivity [65, 66]. In addition, IHC on* in vivo* irradiated human skin showed that a 10-fold increase in the use of HR to repair radiation-induced DNA DSB towards the end of radiotherapy correlated with loss of fractionation sensitivity seen clinically [67]. On average, prostate tumours are thought to have a low alpha/beta ratio and hence are sensitive to both fraction size and total dose of radiation [9]. However, biological heterogeneity means that fraction size sensitivity is likely to vary between prostate tumours and therefore there is a need to identify tissue biomarkers to guide individualised fractionation.

## 3. Biomarkers of Radiosensitivity Identified Using Genomic Techniques

### 3.1. Fluorescent In Situ Hybridisation (FISH)

Use of a fluorogen with FISH, rather than a chromogen as in IHC, means that interpreting expression can be more straightforward than with IHC [68]. In addition, multiple fluorophores can be combined on a single slide which is particularly advantageous with the limited tissue available from pre-radiotherapy biopsies [69]. FISH is routinely available in histopathology laboratories where it is particularly useful for confirming HER2 status in breast tumours and therefore the technique offers considerable potential for development of predictive biomarkers.

FISH has been used to demonstrate a role for biomarkers of cell proliferation such as PTEN and c-myc in predicting radiotherapy response. Loss of the tumour suppressor gene PTEN and amplification of the oncogene c-myc have both been associated with inferior outcomes following radiotherapy [54] (Table 2). In combination, these biomarkers were more strongly associated with increased biochemical recurrence than either in isolation.

### 3.2. Polymerase Chain Reaction (PCR) Array

PCR array involves synthesis and amplification of complimentary DNA (cDNA) prior to expression profiling. Although amplification can introduce bias, this multiplex technique is particularly useful when tissue and hence nucleic acid quantity is limited. A reduction in mRNA (messenger RNA) of the cell-cell adhesion molecule E-cadherin has recently been associated with poor outcome after radiotherapy, but not after primary androgen deprivation therapy alone using PCR array [15]. The authors validated the predictive ability of E-cadherin in an independent dataset by demonstrating that reduced protein expression using IHC was significantly and independently associated with early biochemical recurrence. A number of other candidates were simultaneously assessed including previously discussed EGFR and PTEN, as well as EZH2, PSMA, and MSMB, all of which have shown prognostic ability following surgery; however, they were non-prognostic after radiotherapy in this study. However, the study cohort size was modest at 60 patients which may have contributed to negative findings [15]. The fact that AR also showed no prognostic ability after EBRT illustrates the differences in tumour biology between castration resistant and castration sensitive tumours.

### 3.3. Comparative Genomic Hybridisation (CGH)

CGH differs from Reverse Transcription Polymerase Chain Reaction (RT-PCR) in that it measures DNA copy number variations rather than messenger RNA expression. It thus enables a measure of genomic instability and can calculate the percentage of genomic alteration (PGA) per tumour sample. Today CGH arrays are able to evaluate global copy number variations with as little as 100 ng of DNA [73]. This is highly relevant to localised prostate tumours treated with radiotherapy, not only because there is limited tissue available but also because progression of prostate cancer is known to be characterised by increased chromosomal and subchromosomal alterations characteristic of genomic instability [73]. Some of the earliest prognostic molecular biomarkers identified over two decades ago were based on detection of genomic instability in the form of polyploidy or nondiploidy assessed using flow cytometry and nuclear DNA content measured by static DNA cytometry [52, 53].

Using CGH, copy number loss of several novel biomarkers with diverse functions has been proposed, as well as further validation of previously identified candidates including PTEN [54] (Table 3). These include two genes involved in androgen synthesis, namely, androgen synthesis genes steroidogenic acute regulatory protein (StAR) and hydroxysteroid (17-beta) dehydrogenase 2 HSD17B2 [57]. NKX3.1 is a tumour suppressor gene with a role in prostate stem cell maintenance. It interacts with topoisomerase I and is thought to facilitate recruitment of phosphorylated ATM and gamma H2AX to sites of DNA double strand breaks, both highly relevant to the DNA damage response [55]. NKX3.1 allelic loss alone independently predicted failure from image-guided radiotherapy (IGRT) in a model adjusting for relevant clinical parameters, androgen treatment, radiotherapy dose, and PGA [55]. When allelic gain in c-myc was combined with NKX3.1 loss, the combination showed further predictive capacity [55]. Nibrin (NBN), also known as Nijmegen Breakage Syndrome- (NBS-) 1, forms part of the MRN complex which is central to initiation of the DNA damage response (DDR). In a study of 6 important genes in the DDR (also including MRE11A, RAD50, ATM, ATR, and PRKDC) NBN gain was the only copy number variation significantly predicting biochemical recurrence after IGRT [56]. As it did not predict outcome after radical prostatectomy, NBN may have a role as a predictive biomarker guiding local treatment decisions.

### 3.4. RNA Expression Profiles

A number of RNA expression signatures have been proposed to risk-stratify in localised prostate cancer [74, 75]. The majority have not been evaluated in a radiotherapy cohort due to inadequate tissue quantity, although the oncotype DX for prostate has been tested on needle biopsy specimens [76]. The cell cycle progression score is a 31-gene signature based on RNA expression which was developed using quantitative RT-PCR [77]. The 31 genes were selected from a larger panel of 126 candidate genes known to be involved in cell cycle progression within the Gene Expression Omnibus database. The score includes genes with central roles in DNA repair such as RAD51. Initially developed using radical prostatectomy and TURP specimens [77], the signature has subsequently shown significant prediction of biochemical recurrence following image-guided radiotherapy on multivariate analysis that adjusted for known clinical predictive factors [58].

### 3.5. DNA Signatures

The first known DNA based signature to predict recurrence after EBRT has recently been reported [59] and was developed in a radiotherapy cohort by the use of a customised array to detect copy number alterations together with measurement of partial oxygen pressure using an intraglandular piezoelectrode. Four unique genomic subtypes were identified using unsupervised hierarchical clustering. Information on PGA and hypoxia was then integrated into the genomic subtypes. Finally, supervised machine learning was used to develop a 100-loci 276-gene DNA signature which was validated in a surgically treated cohort. The new signature outperformed a clinical model and 23 previously published RNA signatures in predicting biochemical relapse-free survival. Intriguingly several genes involved in lipid biology were included in the signature; the association of the local cholesterol metabolism of prostate tumours with disease progression has been demonstrated previously [78].

### 3.6. Next Generation Sequencing

Next generation sequencing (NGS) offers enormous potential for personalisation of treatment. It enables assessment of genomic events usually affecting more than 1 kbp such as structural copy number alterations and chromosomal rearrangements including translocations, inversions, and recombination events. In addition, the powerful resolution of NGS also enables detection of events affecting less than 1 kbp such as substitutions, insertions, and deletions [79]. To our knowledge, biomarker signatures using NGS predicting response to radiotherapy are not yet available, and limited tissue availability may be an explanation for this. However, this is likely to change soon with studies involving combined DNA, RNA, and epigenetic analyses ongoing as part of the International Cancer Genome Consortium and The Cancer Genome Atlas [59]. NGS technology continues to evolve rapidly and recently targeted DNA sequencing of prostate tumours using the Illumina platform has been possible with as little as 30 ng of DNA [80] and using the PGM Ion Torrent platform with as little as 6 ng [81].

## 4. Conclusion

Fundamental aspects of cancer biology including DNA repair and hypoxia are intimately related to the efficacy of radiotherapy. It is therefore not surprising that over recent decades a number of promising tissue biomarkers have been developed using a range of molecular techniques. Whilst the majority of biomarker candidates are protein markers developed using IHC, markers of genomic instability using more quantitative techniques have shown excellent prognostic capability. Validation of these biomarkers is a priority so that the added benefit to standard clinical parameters can be clearly quantified and existing inconsistencies resolved. Development of predictive biomarkers that differentiate benefit from different local treatments and active surveillance would further enhance personalised management of early prostate cancer. Challenges include the need for standardised reproducible protocols and antibodies for IHC, together with the technical limitations of using very small biopsies for genomic techniques. However, technology continues to advance rapidly and the potential for molecular biomarkers to improve prediction of both sides of the therapeutic ratio of radiotherapy for localised prostate cancer is hugely promising.

## Figures and Tables

**Table 1:** Predictive tissue biomarkers for radiotherapy response identified using immunohistochemistry (IHC).

Tissue biomarkers for radiotherapy response identified using IHC			
Marker	Function	Technique	IHC cut point used
p53 [22, 23, 25, 27]	Cell cycle checkpoints	IHC	0 versus 1–4^*∗*^ [22], ≤30% nuclei versus >30% nuclei [23], <20% nuclei versus ≥20% nuclei [25], and 0 versus 1 versus 2–4 [27]
p16 [28, 29]	Cell cycle checkpoints	IHC	≤25% versus >25% [28], ≤81.3% versus >81.3% [29]
Rb1 [28]	Cell cycle checkpoints	IHC	≤20% versus >20%
MDM2 [17, 24]	Cell cycle checkpoints	IHC	≤184 versus >184 AU (IA) [17], ≤3% versus >3% ACIS [24]

Ki67 [17–21]	Cell proliferation	IHC	≤11.3% nuclei versus >11.3% nuclei [17], SI ≤3.5% versus >3.5% [18, 19], continuous and SI ≤7.1% versus >7.1% [20], and SI <6.2% versus ≥6.2% [21]
PKA [39]	Cell proliferation	IHC	Manual: 0, 1, and 2 versus 3 and 0, 1 versus 2, 3^*∗*^IA: median of 111.8, Q3 of 128.0 and 135.5
STAT3 (activated) [40]	Cell proliferation/apoptosis	IHC	Continuous and ACIS ≤29% versus >29%

Her2/neu [41]	Growth receptor	IHC	Membrane positivity ≤10% versus >10%
EGFR [42]	Growth receptor	IHC	Negative versus weak or strong membranous staining

Bcl2 [22, 23, 43]	Apoptosis	IHC	Nil versus any cytoplasmic staining [22, 23], ≤20% versus >20% cytoplasmic staining [43]
Bax [44]	Apoptosis	IHC	Greater or lesser cytoplasm staining intensity relative to normal prostate

E-cadherin [15]	Cell adhesion	PCR array, IHC validation	Absent or weak (0/1+) versus moderate or strong (2+/3+)

COX2 [32]	Prostaglandin synthesis	IHC	134 AU (median) and continuous variable [32]

LDH5 [37]	Anaerobic metabolism and hypoxia	IHC	<50% cytoplasmic expression and/or <10% nuclear expression versus >50% and >10%
HIF1a [35, 42]	Hypoxia	IHC	0% versus <1% versus 1–10% versus 10–33% versus 34–67% versus >67% cytoplasmic staining [35], ≤50% versus >50% nuclear and cytoplasm staining [42]
VEGF [35, 36]	Hypoxia	IHC	0% versus <1% versus 1–10% versus 10–50% versus >50% cytoplasmic staining [35], IRS score^*∗*^ 0–4 versus 5–8 [36]

DNA PKcs [45]	NHEJ	IHC	Nil versus any nuclear staining

NHEJ: nonhomologous end joining; PCR: polymerase chain reaction; IA: image analysis; IRS: immunoreactive score; AU: arbitrary units; ACIS: automated cellular imaging system; ^*∗*^cut point refers to semiquantitative scoring system incorporating staining intensity and percentage of tumour cells positive; SI: staining index.

**Table 2:** Predictive tissue biomarkers for radiotherapy response identified using genomic techniques.

Marker/signature	Function	Technique
DNA ploidy [52]	Genomic instability	Image analysis (Feulgen) and DNA/protein flow cytometry
Nuclear DNA content [53]	Genomic instability	Static DNA cytometry

c-myc [54]	Cell proliferation	Array CGH validated by FISH

PTEN [54]	Cell survival	Array CGH validated by FISH

E-cadherin [15]	Cell adhesion	PCR array, IHC validation

NKX3.1 [55]	Androgen related homeobox gene, DNA repair	Array CGH
NBN [56]	DNA damage response	Array CGH

StAR [57]	Androgen synthesis	Array CGH
HSD17B2 [57]	Androgen synthesis	Array CGH

Cell cycle progression score [58]	Cell cycle progression	RT-PCR (RNA expression)

CAN_RF [59]	Genomic and microenvironment heterogeneity	Array CGH, intraglandular hypoxia with piezoelectrode

FISH: fluorescent in situ hybridisation; CGH: comparative genomic hybridisation; RT-PCR: reverse transcription polymerase chain reaction.

**Table 3:** Negative predictive studies in EBRT.

Marker	Function	Technique	IHC cut point used
VEGF [34]	Hypoxia	IHC	0-1 versus 2-3 cytoplasmic staining intensity
Bcl-2 [44, 60, 70]	Apoptosis	IHC	Nil versus any cytoplasmic staining [44, 60], <10% versus >10% cell staining [70]
Bax [60]	Apoptosis	IHC	Greater or lesser cytoplasm staining intensity relative to normal prostate
AR [15]	Androgen receptor	PCR array	
PCA3 [15]	Prostate marker	PCR array	
PTEN [15]	Cell survival	PCR array	
EZH2 [15]	Transcriptional control	PCR array	
EGFR [15]	Growth receptor	PCR array	
PSMA [15]	Prostate marker	PCR array	
MSMB [15]	Tumour suppression	PCR array	
CAG repeats [71]		Genotyping (PCR)	
CYP3A4 polymorphisms [72]		Genotyping	
TMPRSS2/ERG (or ETV1) [16]	Androgen regulated/cell proliferation gene fusion	Array CGH, IHC	Any positive staining versus negative
Osteopontin [46]	SIBLING, tumour associated protein	ELISA	
Ku70 and Ku80 [45]	NHEJ	IHC	Ku70 ≤50% versus >50% nuclear staining, Ku80 ≤60% versus >60% nuclear staining
MRE11 [56]	DNA damage response	Array CGH	
RAD50 [56]	DNA damage response	Array CGH	
ATM [56]	DNA damage response	Array CGH	
ATR [56]	DNA damage response	Array CGH	
PRKDC (DNA PKcs) [56]	NHEJ	Array CGH	

SIBLING: small integrin-binding ligand: N-linked glycoprotein.
